# Key Role of Sequencing to Trace Hepatitis A Viruses Circulating in Italy During a Large Multi-Country European Foodborne Outbreak in 2013

**DOI:** 10.1371/journal.pone.0149642

**Published:** 2016-02-22

**Authors:** Roberto Bruni, Stefania Taffon, Michele Equestre, Paola Chionne, Elisabetta Madonna, Caterina Rizzo, Maria Elena Tosti, Valeria Alfonsi, Lara Ricotta, Dario De Medici, Simona Di Pasquale, Gaia Scavia, Enrico Pavoni, Marina Nadia Losio, Luisa Romanò, Alessandro Remo Zanetti, Anna Morea, Monia Pacenti, Giorgio Palù, Maria Rosaria Capobianchi, Maria Chironna, Maria Grazia Pompa, Anna Rita Ciccaglione, Italian National Task Force on Hepatitis A

**Affiliations:** 1 Department of Infectious, Parasitic and Immune-mediated Diseases, Istituto Superiore di Sanità (ISS), Rome, Italy; 2 Department of Cell Biology and Neurosciences, Istituto Superiore di Sanità (ISS), Rome, Italy; 3 National Centre for Epidemiology, Surveillance and Health Promotion, Istituto Superiore di Sanità (ISS), Rome, Italy; 4 Department of Veterinary Public Health and Food Safety, Istituto Superiore di Sanità (ISS), Rome, Italy; 5 Reparto tecnologia acidi nucleici applicata agli alimenti, Istituto Zooprofilattico Sperimentale della Lombardia e dell’Emilia Romagna, Brescia, Italy; 6 Department of Biomedical Sciences for Health, University of Milan, Milan, Italy; 7 Azienda Ospedaliero-Universitaria Policlinico di Bari, Bari, Italy; 8 Microbiology and Virology Unit, Padua University Hospital, Padua, Italy; 9 Department of Molecular Medicine, University of Padua, Padua, Italy; 10 Laboratory of Virology, Istituto Nazionale per le Malattie Infettive "L. Spallanzani", Rome, Italy; 11 Directorate General for Preventive Health Care, Ministry of Health, Rome, Italy; Oklahoma State University, UNITED STATES

## Abstract

**Background:**

Foodborne Hepatitis A Virus (HAV) outbreaks are being recognized as an emerging public health problem in industrialized countries. In 2013 three foodborne HAV outbreaks occurred in Europe and one in USA. During the largest of the three European outbreaks, most cases occurred in Italy (>1,200 cases as of March 31, 2014). A national Task Force was established at the beginning of the outbreak by the Ministry of Health. Mixed frozen berries were early demonstrated to be the source of infection by the identity of viral sequences in patients and in food. In the present study the molecular characterization of HAV isolates from 355 Italian cases is reported.

**Methods:**

Molecular characterization was carried out by PCR/sequencing (VP1/2A region), comparison with reference strains and phylogenetic analysis.

**Results:**

A unique strain was responsible for most characterized cases (235/355, 66.1%). Molecular data had a key role in tracing this outbreak, allowing 110 out of the 235 outbreak cases (46.8%) to be recognized in absence of any other link. The data also showed background circulation of further unrelated strains, both autochthonous and travel related, whose sequence comparison highlighted minor outbreaks and small clusters, most of them unrecognized on the basis of epidemiological data. Phylogenetic analysis showed most isolates from travel related cases clustering with reference strains originating from the same geographical area of travel.

**Conclusions:**

In conclusion, the study documents, in a real outbreak context, the crucial role of molecular analysis in investigating an old but re-emerging pathogen. Improving the molecular knowledge of HAV strains, both autochthonous and circulating in countries from which potentially contaminated foods are imported, will become increasingly important to control outbreaks by supporting trace back activities, aiming to identify the geographical source(s) of contaminated food, as well as public health interventions.

## Introduction

Hepatitis A Virus (HAV) is a member of the Picornaviridae family, genus Hepatovirus. On the basis of genome sequence divergence, all viruses infecting humans have been classified in three genotypes (I, II, III), further divided into two sub-genotypes (A and B) [1]. Genotypes and subtypes show different geographic distribution [2].

HAV is mainly transmitted by the fecal-oral route, e.g., through ingestion of contaminated water and food or close contacts with infected subjects [2].

The infection is usually asymptomatic or mild in children under five years, while in adults more frequently occurs with symptoms and jaundice. Clearance of infection confers lifelong immunity. In highly endemic countries, HAV infection is acquired in early childhood and most adult population is positive for anti-HAV IgG and protected from reinfection. In contrast, in low endemicity countries, most adult population is susceptible: as a result, infections are more likely to occur in adults, in which are frequently symptomatic, and foodborne outbreaks are increasingly reported [3].

Three different foodborne HAV outbreaks occurred in Europe and one in USA in 2013 and the vehicle of infection in all of them was a minimally processed vegetable product, in particular fresh and frozen strawberries, mixed frozen berries and pomegranate seeds [4,5]. These outbreaks were associated with specific HAV strains, supporting molecular typing as a key tool for early outbreak identification, by discovering potential links between apparently unrelated cases, even those occurring in different countries [6].

The largest of the multi-country European HAV outbreaks involved 1,315 total cases as of March 31, 2014, with most cases being observed in Italy (n = 1,202) [7]. Local Health Services (LHS) were promptly alerted by the Ministry of Health, that established an enhanced surveillance including virus genotyping. Early epidemiological and laboratory investigations linked the outbreak to mixed frozen berries [8–10].

In the present study the molecular characterization of 355 HAV isolates from Italian cases is reported. Sequencing was crucial both to identify outbreak cases and to highlight unrelated minor outbreaks and small clusters, providing an overview of the viral strains circulating in Italy during the multi-country European epidemic.

## Materials and Methods

### Samples and Sequences

In May 2013 a specific alert was issued by the Italian Ministry of Health, recommending the LHS to enhance hepatitis A surveillance as well as to send clinical samples to the National Reference Laboratory for Hepatitis Viruses at Istituto Superiore di Sanità (NRL-ISS) or to Regional laboratories, for viral characterization. This study was done in response to a public health emergency and was thus exempt from institutional review board approval. All data were analyzed anonymously.

The study population included 355 out of the 1,202 total cases notified throughout Italy to the Italian surveillance system with onset from January 1, 2013 to February 28, 2014. In particular, the study population included all cases from which a HAV sequence could be obtained from a biological sample sent by infectious disease hospitals either to the NRL-ISS or to Regional laboratories. Information about travel history, known for 287 cases, showed that the vast majority of them did not exhibit the "travel to endemic areas" risk factor: 215 cases (74.9%) reported no travel outside Italy and, among the remaining 72 cases, 14 (4.9%) travelled to an EU country, 32 (11.1%) outside EU, 26 (9.1%) did not specify the destination country in the questionnaire.

The NRL-ISS received: (a) sera from nine Italian regions (Valle d’Aosta, Piedmont, Lombardy, Emilia Romagna, Tuscany, Marche, Umbria, Latium, Sardinia) and two autonomous provinces (Trento and Bolzano), leading to 108 HAV sequences; (b) sequences from five Regional laboratories corresponding to 72 cases from Lombardy, 49 from Emilia Romagna, 69 from Apulia, 35 from Veneto, 22 from Latium. Overall, sequences from 355 cases from 11 Italian regions and two autonomous provinces and with onset from January 1, 2013 to February 28, 2014 were finally included in the national sequence database at ISS for the analysis. Demographic data and risk factors were available through the National Surveillance System for Acute Hepatitis (SEIEVA), which collects notifications reporting information obtained from patients by a standard questionnaire.

### Viral RNA Extraction, Reverse Transcription and Nested PCR of Sera Analyzed at ISS

Viral RNA was extracted from 140 μl serum by using the QIAmp viral RNA extraction kit (Qiagen, Hilden, Germany). One sixth extracted RNA (10 μL) was reverse transcribed by the SuperScript III First-Strand Synthesis System for RT-PCR (Invitrogen) with random hexamers.

To obtain sequence information exactly from the same HAV genome region (VP1/2A) sequenced in other European laboratories, nested PCR and sequencing were carried out by using primers reported in a protocol kindly provided by Dr. Linda Verhoef, RIVM, Bilthoven, The Netherlands [11]. Although this protocol based on VP1/2A sequencing provides more limited molecular information than protocols based on larger sub-genomic regions or the full-length HAV genome, it was preferred for several reasons: (a) the main aim of molecular characterization was the timely monitoring of the ongoing outbreak: double strand sequencing of a single PCR fragment is easy and fast; (b) the cost is lower than sequencing larger genomic regions; (c) VP1/2A sequencing is the most commonly investigated region in outbreaks: a large number of previously sequenced strains are available for comparison in public databases; (d) preliminary VP1/2A sequence data obtained early in the outbreak showed that information was sufficient to discern “outbreak” and “non outbreak” cases; (e) the present outbreak involved several European countries (cases were finally identified over 11 countries): both at the beginning of the outbreak and later on, there was a need for timely comparison of the viral sequences from different countries, to understand if a common multi-state outbreak or different independent outbreaks were ongoing. Some countries were already using protocols based on VP1/2A sequencing, so the European Center for Disease Control (ECDC) highly recommended all involved member states to sequence that viral region, to allow easy and fast comparison of the strains responsible for hepatitis A cases in different countries.

### Sequencing

Double strand sequencing of purified PCR products obtained from human sera was carried out by using the GenomeLab DTCS Quick Start KiT and an automated DNA sequencer (Beckman Coulter, Inc., Fullerton, CA). The sequenced region encompassed the VP1/2A region of HAV genome (460 nt, positions 2915 to 3374 in the HM-175 reference sequence Acc.No. NC_001489).

The NRL-ISS also received sequences, spanning the VP1/2A region, from five Regional laboratories. One laboratory (IZSLER—Emilia Romagna) carried out sequencing with the same protocol used at ISS. The four other laboratories used different protocols and, thus, sent sequences of variable size. A laboratory (UNIMI—Lombardy) sent both “long” (439 to 457 nt) and “short” sequences (270 to 349 nt), depending on whether a PCR product suitable for sequencing was obtained just at the first PCR step or required a nested PCR amplification (producing "long" or “short” sequences, respectively). Another laboratory (A.O.U. Bari—Apulia) did not send sequences from individual cases, but sent four sequences representing the four strains identified in 39, 11, 1 and 18 cases; these sequences overlapped the region sequenced at ISS for 460, 338, 358 and 349 nt, respectively. A fourth laboratory (INMI—Latium) sent sequences whose size was 225 nt, and a fifth laboratory (A.O.PD—Veneto) sent sequences ranging from 226 to 247 nt.

Alignment of the 225 to 358 nt sequences showed they shared a 174 nt common region, (positions 2967 to 3140 in the HM-175 reference sequence, Acc.No. NC_001489) suitable to compare them.

All sequences were included in two different datasets according to their sequence length (Dataset 1: 218 sequences, 419 to 460 nt; Dataset 2: included the 174 nt region shared by 137 sequences whose original size was 225 to 358 nt) and analyzed separately according to the rules described in the next paragraph. Accession numbers are reported in S1 Appendix.

### Sequence Analysis

Sequence analysis was performed at the NRL-ISS. Genotype was assigned by phylogenetic analysis (Neighbour-Joining) with reference sequences (see S2 Appendix).

To search for cases specifically due to the “outbreak” strain, the sequences from human sera were compared to the reference sequence Acc. No. KF182323 [8]. It was the first sequence obtained by the NRL-ISS on May 2013 from an Italian case associated with consumption of frozen berries, and had shown 100% identity both with the sequence obtained from a batch of frozen berries (Acc.no. KF773842) and with the sequences from German and Dutch tourists who got infected some weeks earlier in North-Eastern Italy [8].

In agreement with other EU member states and approval by the ECDC, different criteria were established to consider a sequence as “outbreak”, depending on its length. Sequences 419 to 460 nt were considered to be “outbreak” if 100% identity or, at most, 1 nt difference over the entire length was observed. A more restrictive rule was applied to the shorter sequences, considered to be “outbreak” exclusively if 100% identity was shown over the 174 nt common region.

To investigate the phylogenetic relationships with strains from endemic and non-endemic countries available in GenBank, a third dataset (Dataset 3) was built including all collected sequences sharing at least a 330 nt region. This size was selected as a balance between the need to retain as much as possible molecular information for reliable analysis and the need to include as much as possible sequences. The dataset also included the reference strains used for genotyping (S2 Appendix) and additional strains retrieved from GenBank according to the following procedure: each sequence identified in cases was used as input in BLAST search to detect sequences in GenBank showing >98% identity over the 330 nt region of interest. Sequences for which the country of infection was available (reported either in the sequence annotations or in a published paper) were then retrieved and included in the dataset.

Phylogenetic relationships were analyzed by constructing Maximum Parsimony trees by MEGA 6.0 [12]. Tree reliability was assessed by setting bootstrap to 1000. Bootstrap values > 70 were considered significant.

## Results

### Identification of “Outbreak” Cases by Sequence Analysis

Two different datasets were built to identify outbreak cases by comparison with the reference Italian outbreak sequence (Acc.no. KF182323). Dataset 1 included 218 sequences whose length was at least 419 nt, Dataset 2 included a 174 nt common region shared by 137 sequences whose length was 225 to 358 nt.

Analysis of Dataset 1 showed that most cases were caused by the “outbreak” strain (142/218, 65.1%) including both sequences showing 100% identity (n = 127) and closely related sequences with no more than 1 nt difference (n = 15) (see Materials and Methods).

**Fig 1:**
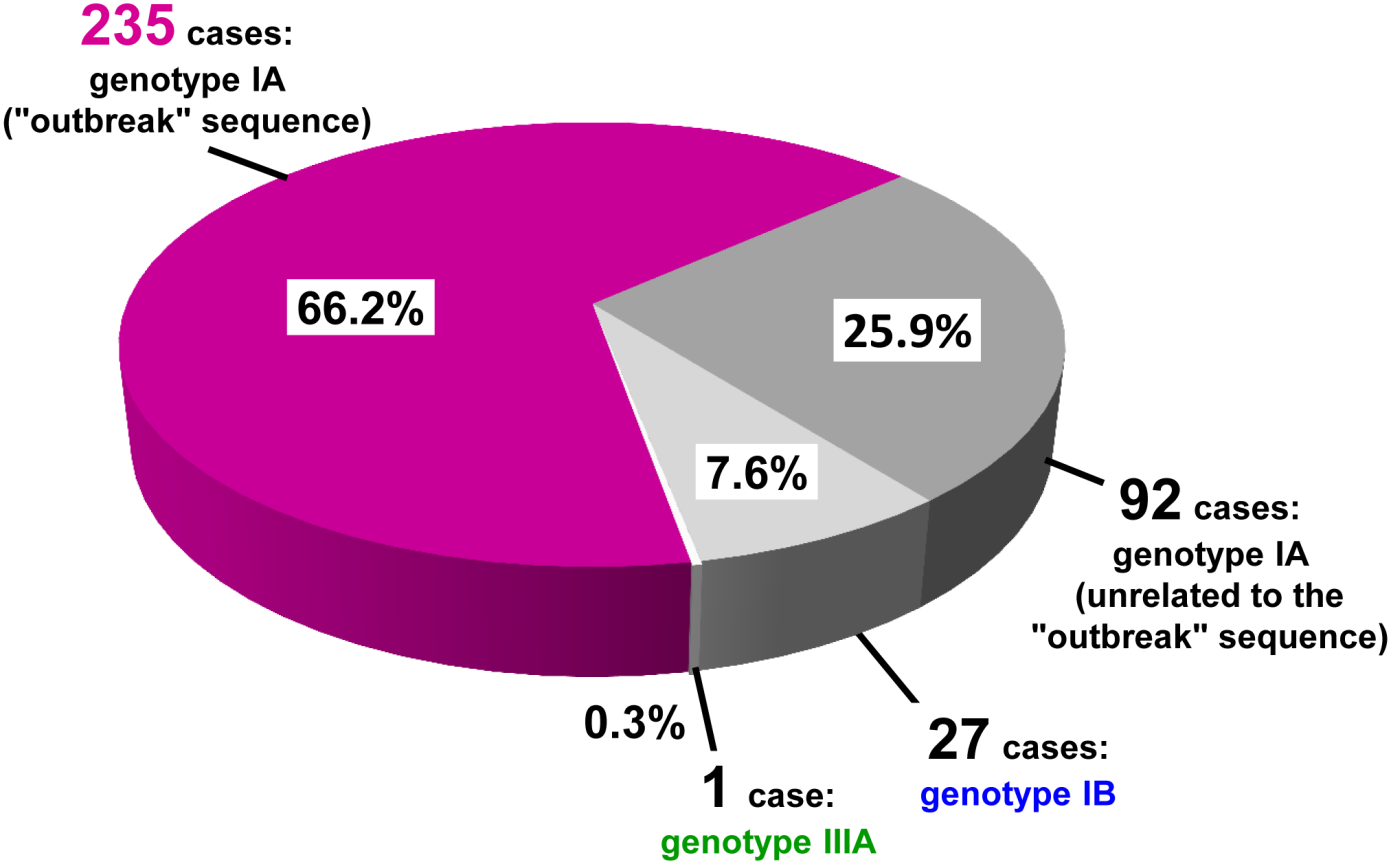
Genotype distribution of HAV sequences (n = 355) from cases with clinical onset between January 1, 2013 and February 28, 2014. The fraction of genotype IA isolates with the mixed frozen berry “outbreak” sequence is reported in purple.

To evaluate if the small 174 nt region in Dataset 2, coupled to the more restrictive criterium of complete identity with the reference sequence, could reliably identify outbreak cases, sequences from Dataset 1 were re-classified limiting analysis to the 174 nt portion. Only 1 out of 218 (0.6%) “long” sequences (including 142 outbreak and 76 unrelated IA, IB and IIIA sequences) was mis-classified: it was a sequence showing 3 nt differences over the entire length (442 nt) but no differences over the 174 nt region. This result supported the reliability of the 174 nt region for correct “outbreak” and “non-outbreak” classification of the 137 “short” sequences. Thus, 355 cases could be finally classified (Fig 1).

From January 1, 2013 to February 28, 2014, 235 cases (66.2%) harbored an HAV isolate with the “outbreak sequence”, 92 cases (25.9%) harbored unrelated genotype IA sequences (92.3% to 97.1% identity with the reference), 27 cases (7.6%) had genotype IB and 1 case (0.3%) was infected by a genotype IIIA virus (Fig 1).

Importantly, 110 out of the 235 “outbreak” cases (46.8%) could be recognized only by virtue of sequence identity, because consumption of frozen berries was either denied or not reported in their questionnaires.

### Variability of the Outbreak Strain

Among the 15 outbreak cases showing a single nucleotide difference *versus* the reference, two clusters (4 and 5 sequences each) could be recognized, each of them containing sequences with identical nucleotide difference (Table 1, in bold). Each of the remaining 6 sequences showed a unique nucleotide difference, leading to a predicted amino acid change in 3 of them (Table 1).

**Table 1:** Summary of the 15 sequences showing one only nucleotide difference *vs*. the reference outbreak sequence (Accession Number KF182323).

Sequence ID	Region or A.P.* of the case	Onset date	nt^†^ position^‡^	nt change	Codon position	aa^§^ change
ISS_82_95	Tuscany	06.13.2013	31	T → C	3^rd^	-
ISS_9_20	A.P. Trento	05.03.2013	349	T → C	3^rd^	-
**UNIMI-ID132/45**	**Lombardy**	**05.31.2013**	**358**	**G → A**	**3**^**rd**^	-
**UNIMI-ID132/42**	**Lombardy**	**06.07.2013**	**358**	**G → A**	**3**^**rd**^	-
**ISS_48_58**	**Marche**	**06.28.2013**	**358**	**G → A**	**3**^**rd**^	-
**UNIMI-ID132/68**	**Lombardy**	**07.06.2013**	**358**	**G → A**	**3**^**rd**^	-
IZSLER_4081_14	Emilia Romagna	12.01.2013	442	T → C	3^rd^	-
**UNIMI-ID132/60**	**Lombardy**	**06.19.2013**	**457**	**T → C**	**3**^**rd**^	-
**UNIMI-ID132/62**	**Lombardy**	**06.28.2013**	**457**	**T → C**	**3**^**rd**^	-
**UNIMI-ID132/65**	**Lombardy**	**06.28.2013**	**457**	**T → C**	**3**^**rd**^	-
**UNIMI-ID132/64**	**Lombardy**	**07.01.2013**	**457**	**T → C**	**3**^**rd**^	-
**UNIMI-ID132/125**	**Lombardy**	**10.01.2013**	**457**	**T → C**	**3**^**rd**^	-
ISS_98_113	A.P. Trento	07.04.2013	127	A → T	3^rd^	R → S
ISS_99_114	A.P. Trento	07.02.2013	372	A → T	2^nd^	K → I
IZSLER_159915_13	Emilia Romagna	06.01.2013	434	G → C	1^st^	A → P

*A.P.: Autonomous Province.

^†^nt: nucleotide.

^‡^position in the reference outbreak sequence Accession Number KF182323 (460 nucleotides).

^§^aa: amino acid.

In bold: two clusters of sequences, each of them sharing the same nucleotide change in the same nucleotide position.

One additional sequence (UNIMI-ID132/49) showed 3 nucleotide differences over 442 nt (99.3% identity). Although this isolate could not be classified as “outbreak” strain, it was obviously much more correlated to it than to the other genotype IA isolates (these latter showing 20–35 nucleotide differences *vs*. the reference).

### Geographical and Temporal Distribution of the Cases in Italy during the Outbreak

**Fig 2:**
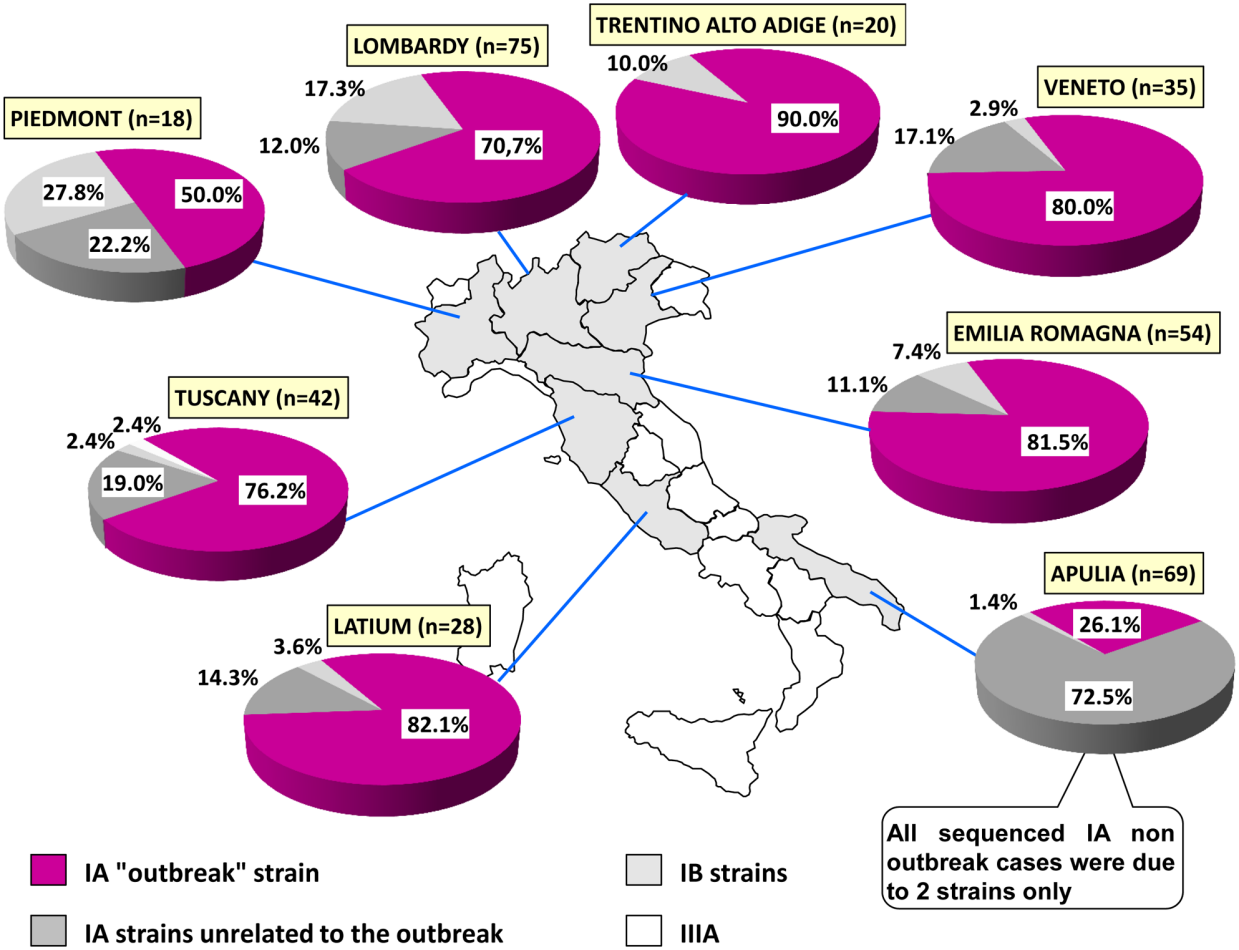
Distribution of HAV strains in the 8 Italian Regions for which enough sequences were available.

Fig 2 reports the distribution of sequences from 8 Italian regions, representing 96% of the collected samples.

The outbreak strain exceeded two-thirds of cases (range: 70.7–90%) in 6 out of 8 regions, except Piedmont and Apulia. In Piedmont a cluster of 4 cases from a Roma camp (sharing an identical strain, genotype IB) strongly affected the distribution, because of the small sample size (18 total sequences). In Apulia, 72.5% of cases were caused by two autochthonous genotype IA strains and only 26% by the “outbreak” strain. The tradition of eating raw mussels in Apulia represents the main risk factor associated with the two autochthonous strains, explaining the peculiar distribution.

**Fig 3:**
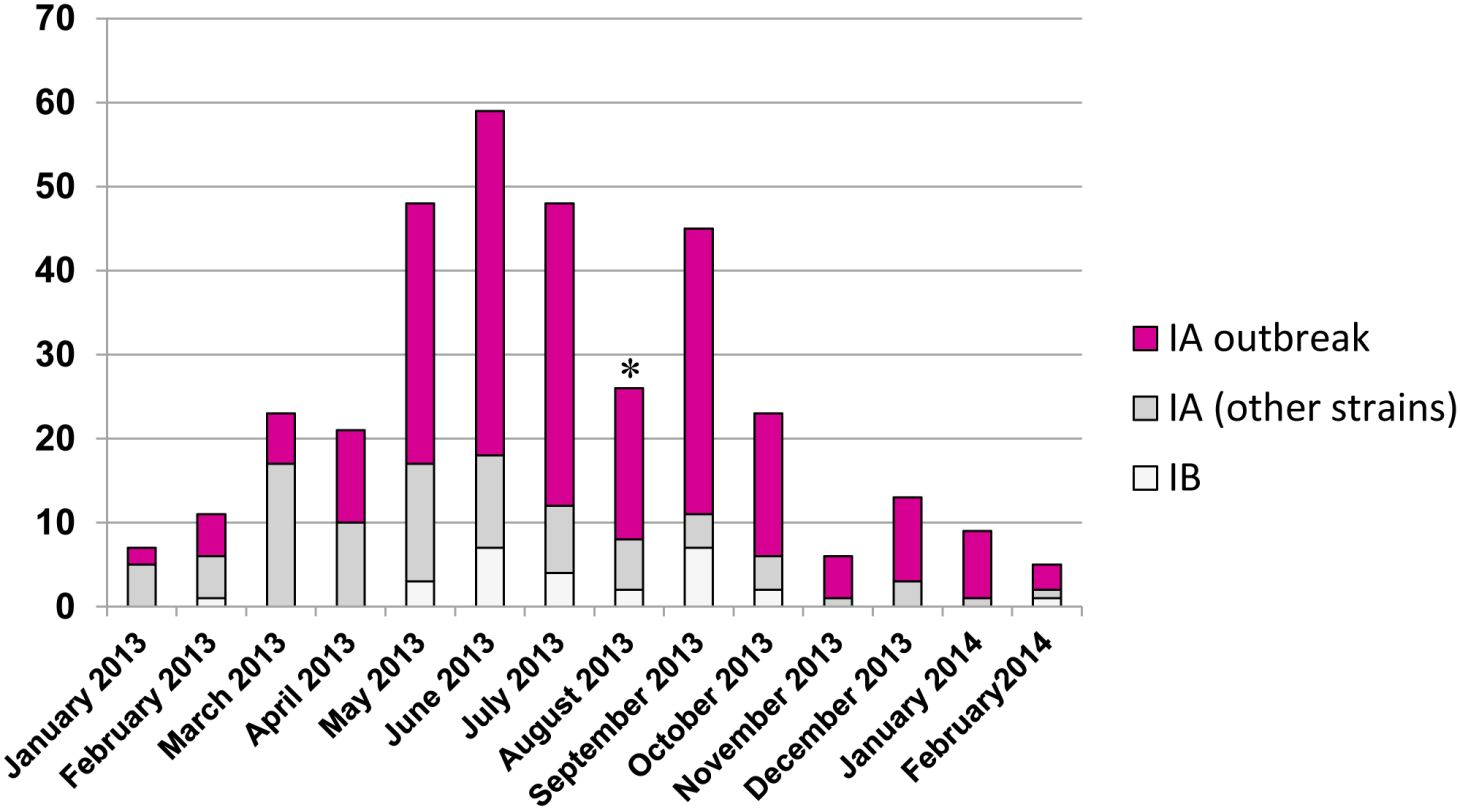
Temporal distribution of the strains, based on the date of symptom onset. * the lower number of cases in August is likely the result of under-delivery of samples, because the trend of notified cases did not show a parallel decrease to a similar extent.

Fig 3 reports the temporal distribution of sequenced cases by onset date. The outbreak strain prevailed in many months. From January through April, most sequenced cases were from Apulia, the only Italian region in which virological surveillance by sequencing was routinely performed: so the distribution of outbreak and non-outbreak strains in those months substantially represented the distribution in Apulia (see Fig 2). A steady increase of sequenced cases could be observed since May, because of the alert issued by the Ministry of Health. Then, the monthly amount of sequenced isolates was proportional to the amount of notified cases (data not shown). The temporal distribution also documents a remarkable circulation of non-outbreak strains throughout the observation period.

### Analysis of Risk Factors in Sequenced Cases

**Fig 4:**
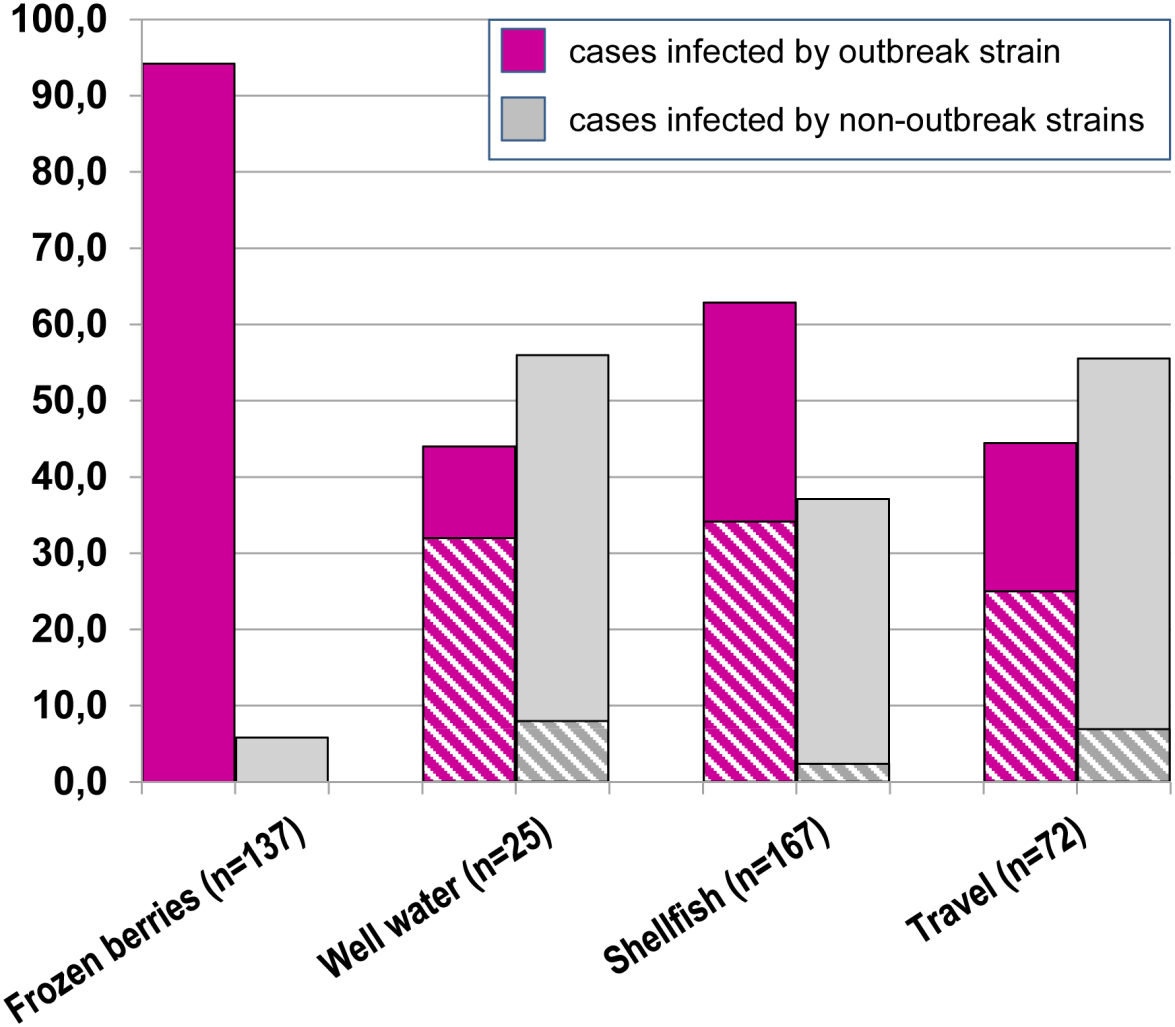
Sequence type (“outbreak” or “non-outbreak”) associated to the risk factors reported in questionnaires by interviewed patients. Multiple risk factors could be reported by each patient. In the “well-water”, “shellfish” and “travels” (outside Italy) groups the proportion of patients also reporting “frozen berries” is highlighted (striped region).

Fig 4 reports the distribution of outbreak and non-outbreak strains in association with consumption of frozen berries (or related foods) and other risk factors.

In the "mixed frozen berries" group, most cases had been infected by the outbreak strain (129/137, 94.2%), although only 36% of them showed exclusively this risk factor.

A high frequency of the outbreak strain was also observed in the other groups (Fig 4, “well water”, “shellfish” and “travels”). This result can be partly explained by multiple risk factors being frequently reported: over half of the cases infected by the outbreak strain also referred consumption of frozen berries (“well water”: 8/11 cases; “shellfish”: 57/105 cases; “travels”: 18/32 cases) (Fig 4, striped portion of the bars). However, a remaining fraction of cases with the outbreak sequence, but not reporting consumption of frozen berries, was also observed (65/277, 23.5%). The presence of the outbreak sequence in these cases may be explained by (a) cases occurred before the alert issued by the Ministry of Health (question about consumption of berries still lacking in the questionnaire), (b) recall bias, (c) unaware consumption or (d) transmission by other routes.

### Phylogenetic Analysis of the Strains Circulating during the Outbreak

S1 Fig shows the results of phylogenetic analysis of the sequences circulating during the outbreak. The sequence dataset (Dataset 3) included: 1 sequence of the strain responsible for the “mixed frozen berry outbreak" (detected in 235 cases), 57 sequences unrelated to the "outbreak" sequence (detected in 105 cases) and 47 reference strains (see Materials and methods). Four main sub-trees may be recognized, corresponding to the IA and IB sub-genotypes and the II and III genotypes (S1 Fig, left upper panel, colored sections).

In the IA sub-tree, two main statistically supported clades can be observed (S1 Fig, enlarged IA section, clade 1 and 2).

Clade 1 included 21 sequences from the present study: most of them (12 isolates) were from Moroccan patients, 10 of which reporting travel to Morocco. The median age of the Moroccan patients was 6 years (range 2–15 years). A sub-cluster of 7 identical sequences can be observed (S1 Fig, red sequences): the sequences were from 4 Moroccan children and 3 Italian adults. Four cases occurred in a same province of Tuscany and in a relatively short timeframe (5 April to 24 July), suggesting a local outbreak; no other link could be found for the remaining 3 cases. Another small sub-cluster (3 cases) was observed in a town in Piedmont (S1 Fig, enlarged IA section, blue sequences): sequences grouped with a reference sequence from a Tunisian patient. The first case (ISS_32_40) was an adult reporting a travel to Tunisia, the two other cases were children.

Clade 2 included 11 sequences from the present study: three of them represent the three strains identified, respectively, in 235 cases (ISS_2_2, mixed frozen berries outbreak 2013), 11 cases (A.O.U._BA_MAN-BA/2013, from Apulia) and 39 cases (A.O.U._BA_SCA-BAT/2013, from Apulia) (S1 Fig, enlarged IA section).

The strain responsible for the mixed frozen berry outbreak in 2013 was previously reported to be responsible for an outbreak in the Czech Republic in 2008 [13;14]. In the tree, it clustered with Venezuelan strains sampled in 2005–2006 (S1 Fig) (only the two most closely related of seven Venezuelan isolates were reported in the phylogenetic tree). This relationship with Venezuelan strains was also observed previously, based on comparison of 225 nt sequences [15]. The nucleotide identity actually proved to be quite less extensive when the longer 460 nt sequence was compared: the outbreak sequence showed 1 to 3 nt differences over 225 nt (98.7–99.6% identity), but 5 to 11 nt differences over 460 nt (97.6–98.9% identity). This observation highlights the importance of sequencing an as large as possible region to better discriminate closely related strains The identity level was also confirmed over a larger region (13 to 29 nt differences over 1,138 nt, 97.5–98.9%) by comparison of Venezuelan strains with the full-length genome sequence of the outbreak strain available from GenBank, obtained from *in vitro* cultured HAV originating from a sample of mixed frozen berries [16].

The two strains from Apulia in clade 2 grouped with isolates circulating in that Italian region in 2008 and 2011 (KF706400, KF233556, KF706398) and with references from Russia. Two Romanian cases from Tuscany and Emilia Romagna (ISS_113_131 and IZSLER_5207_14), clustering in the same group, showed no apparent epidemiological link with the other isolates, despite 100% sequence identity.

Although not all nodes were statistically supported, most remaining sequences grouped with references from the country of origin of the cases or from the same geographical area of travel (Ecuador, Brasil, Indonesia, Philippines).

In the IB sub-tree, sequences grouped in two main clades (S1 Fig, enlarged IB section, clades 3 and 4). Clade 3 included sequences from 7 cases: 4 of them reported having travelled to Morocco. The median age of the 7 cases was 12 years (range 10–19 years). Two reference sequences, from North Africa and from a patient travelling to Morocco, also clustered in this group.

Clade 4 included 17 cases: 7 of them reported travel to African countries (Kenya, Madagascar, Namibia, Egypt) and in most cases their sequences were phylogenetically related to references from Africa. Two cases, occurred in Tuscany and Emilia Romagna, showed an identical IB strain and were associated with travel to Namibia. An “Egyptian” group can also be observed, although did not show bootstrap support.

Finally, a statistically supported cluster unrelated to travel included 4 cases with identical sequences, 3 of them living in a Roma camp in Piedmont. Their median age was 7 years (range 5–8 years).

No genotype II cases were observed. The genotype III sub-tree included the only one case observed in the studied period as well as an additional case occurred a few days later (S1 Fig, left upper panel); both cases were travel related.

## Discussion

The complete identity between the outbreak sequence from human cases and the sequence from a batch of frozen berries, a finding infrequently reported in foodborne HAV outbreaks, strongly suggested frozen berries as the common source since the beginning of the epidemic [8].

Analysis of 355 cases showed that a unique HAV strain was responsible for two thirds of them. As the sequencing was carried out on a high proportion of cases notified in the same time frame (355 of 1,202 notified cases, 29.5%), the distribution of viral strains in the sequenced cases may roughly represent the distribution in the notified cases. Thus, the “outbreak” strain seems to be responsible for most excess notifications occurred in the studied period.

The strain had been already involved in a large outbreak in the Czech Republic in 2008, with 1616 reported cases [13,14]. However, the Czech outbreak was not foodborne: it started among intravenous drug users, continued in other high-risk groups, then spread in the general population [13]. Since then, no further cases due to this strain were reported in Europe until the 2013 outbreak.

The strain is phylogenetically related to Venezuelan isolates sampled in 2005–2006. Although this finding suggests a possible common evolutionary ancestor, this hypothesis cannot yet be formally proved on the basis of available data. In addition, no conclusions about the possible direct origin of the Czech strain from Venezuelan strains or *vice versa* can be drawn, as the several nucleotide differences imply a long-lasting evolution and a possible mutual migration between continents. So, the phylogenetic and geographical origin of the outbreak strain remains an open question.

The present investigation highlights, once again, the molecular characterization as an essential tool to trace outbreaks. In fact, molecular typing had a crucial role for identification of epidemic cases lacking any epidemiological link: 110 out of 235 confirmed cases (46.8%) could be correctly classified only by virtue of molecular data.

Although the outbreak strain could be reliably identified also by restricting the analysis to a small region of 174 nt, closely related variants could be discerned only by analyzing longer sequences. These variants could be either the result of random variation during person-to-person transmission or the result of food contamination at production points, in which multiple related variants might circulate. The finding of two clusters of variants, each marked by a specific nucleotide variation, in cases with no apparent link supports this latter hypothesis. Recognizing variants would be valuable for tracing the origin of contaminated food by comparison with strains from endemic areas, whose better knowledge appears to be increasingly necessary.

Interestingly, molecular characterization also revealed the circulation of other unrelated strains, some of them responsible for minor outbreaks and small clusters (2 to 39 cases), the smallest of which had not been recognized on the basis of epidemiological data.

In Apulia, 50 hepatitis A cases linked to shellfish consumption were demonstrated to be caused by two distinct IA strains, circulating simultaneously and responsible for 11 and 39 cases each (S1 Fig). The two strains appeared to be phylogenetically related to Russian strains as well as to local strains detected in previous years. Eating raw shellfish is very popular in Apulia and is responsible for several cases each year.

Among the small clusters, the largest of them included 7 cases due to a IA strain likely originating from Morocco, on the basis of phylogenetic data; only 2 out of 7 cases reported “travel to Morocco”, suggesting local transmission in Italy in the remaining cases.

Analysis of sporadic cases showed that in 2013 most of them were linked to travel to endemic countries, Morocco being the most frequently reported. Importantly, available data suggested that in several cases the involved strains, both IA and IB, had been imported by Moroccan children/adolescents travelling to Morocco, but were then transmitted to other children/adolescents, mainly with Moroccan nationality, in Italy. The majority of the remaining travel related cases occurred in adults and their viral sequence revealed phylogenetic relationship to strains circulating in the same country/area of travel.

## Conclusions

In conclusion, the outbreak occurred in 2013 prompted Italy to implement molecular surveillance of HAV. In addition to tracing the outbreak, the surveillance provided an overview of the strains circulating in Italy and led to establish a national database of autochthonous and imported HAV sequences that will be helpful in the next years, especially to support trace-back activities in identifying the geographical source(s) of contaminated food. However, important gaps still remain in the molecular knowledge of HAV strains circulating in those Europe and extra-Europe countries in which no molecular surveillance is established and from which potentially contaminated foods are imported. Eliminating these gaps will require improved international collaboration and data exchange, to fight emerging HAV outbreaks.

## Supporting Information

S1 Appendix Accession number of the sequences obtained from hepatitis A cases reported in the present study. (DOCX)

S2 Appendix Reference sequences for genotyping. (PDF)

S1 Fig Phylogenetic tree of 58 sequences representative of 340 cases occurred from January 1, 2013 to February 28, 2014 and 47 reference sequences. The tree was obtained by the Maximum Parsimony approach. The 58 sequences from the present study are reported in bold: 1 of them (reported in purple color) is representative of 235 cases of the mixed frozen berries outbreak; 2 other IA sequences represent 11 and 39 cases, respectively; the remaining 55 sequences are from individual cases. The IA and IB subtrees are shown enlarged. For each sequence from the present study the following information is reported, wherever available, in the following order: ID code, Italian region of the patient, nationality for non-Italian patients (none indicated if Italian), risk factor(s) (be, ww, sh, trv., in parentheses), age group (child: 0–12 years; teenager: 13–19 years; adult: >19 years), date of clinical onset (only for some relevant cases). For three IA sequences (ISS_2_2 PA TRENTO KF182323, A.O.U.BA MAN-BA/2013 and A.O.U.BA SCA-BAT/2013) the number of cases in which the sequence was found is also reported. For each reference sequence, information is reported in the following order: accession number, sequence name, country of isolation, risk factor(s) (if known), year of isolation, genotype/sub-genotype. Some reference sequences were from vegetable matrices (dates and orange juice, indicated). The four main clades in the genotype IA and IB sub-trees are labeled “1” to “4”. Two clusters in clade 1 are highlighted by red and blue fonts, respectively (see the main text for details). Significant bootstrap values are reported.
(PDF)
