# Supplementary material for: Key Role of Sequencing to Trace Hepatitis A Viruses Circulating in Italy During a Large Multi-Country European Foodborne Outbreak in 2013
Source: PLoS One. 2016 Feb 22;11(2):e0149642. doi: 10.1371/journal.pone.0149642 (PMC4764681; doi:10.1371/journal.pone.0149642)
Supplement: S1 Appendix — (DOCX) [file pone.0149642.s001.docx]

**S1 Appendix. Accession number of the sequences obtained from hepatitis A cases reported in the present study.**

The sequences reported in the present study were deposited in GenBank under the following Accession Numbers: KF182323 (“outbreak strain”, representative of the outbreak cases showing identical sequence); from KU570221 to KU570292; from KF475774 to KF475780; from KF706406 to KF706408; KJ614459; KJ614461; KJ614462; KJ614464; KJ614467; KJ614469: KJ614470; KJ614473; KJ614475; KJ614477; KJ614479; KJ614481; KJ614482.
