# Supplementary material for: Key Role of Sequencing to Trace Hepatitis A Viruses Circulating in Italy During a Large Multi-Country European Foodborne Outbreak in 2013
Source: PLoS One. 2016 Feb 22;11(2):e0149642. doi: 10.1371/journal.pone.0149642 (PMC4764681; doi:10.1371/journal.pone.0149642)
Supplement: S2 Appendix — (PDF) [file pone.0149642.s002.pdf]

S2 Appendix. Reference sequences for genotyping. The following reference sequences were used for genotyping by phylogenetic analysis: genotype IA: X75215 (GBM/WT strain, Germany), X83302 (FG strain, Italy); genotype IB: NC_001489 (HM-175 strain, Australia); AF314208 (L-A-1 strain, China); M20273 (MBB strain, North Africa); AF268396 (HAF-203 strain, Brasil); KC876797 (T34213 strain, Denmark_2013); genotype IIA: AY644676 (CF53/Berne strain, France); genotype IIB: AY644670 (SLF88 strain, Sierra Leone); genotype IIIA: AJ299464 (NOR-21 strain, Norway); KF706410 (HAV/GIU/BA/2012/IT, Italy); genotype IIIB: AB258387 (HA-JNG06-90F, Japan).
