# Supplementary material for: Key Role of Sequencing to Trace Hepatitis A Viruses Circulating in Italy During a Large Multi-Country European Foodborne Outbreak in 2013
Source: PLoS One. 2016 Feb 22;11(2):e0149642. doi: 10.1371/journal.pone.0149642 (PMC4764681; doi:10.1371/journal.pone.0149642)
Supplement: S1 Fig — The tree was obtained by the Maximum Parsimony approach. The 58 sequences from the present study are reported in bold: 1 of them (reported in purple color) is representative of 235 cases of the mixed frozen berries outbreak; 2 other IA sequences represent 11 and 39 cases, respectively; the remaining 55 sequences are from individual cases. The IA and IB subtrees are shown enlarged. For each sequence from the present study the following information is reported, wherever available, in the following order: ID code, Italian region of the patient, nationality for non-Italian patients (none indicated if Italian), risk factor(s) (be, ww, sh, trv., in parentheses), age group (child: 0–12 years; teenager: 13–19 years; adult: >19 years), date of clinical onset (only for some relevant cases). For three IA sequences (ISS_2_2 PA TRENTO KF182323, A.O.U.BA MAN-BA/2013 and A.O.U.BA SCA-BAT/2013) the number of cases in which the sequence was found is also reported. For each reference sequence, information is reported in the following order: accession number, sequence name, country of isolation, risk factor(s) (if known), year of isolation, genotype/sub-genotype. Some reference sequences were from vegetable matrices (dates and orange juice, indicated). The four main clades in the genotype IA and IB sub-trees are labeled “1” to “4”. Two clusters in clade 1 are highlighted by red and blue fonts, respectively (see the main text for details). Significant bootstrap values are reported. (PDF) [file pone.0149642.s003.pdf]

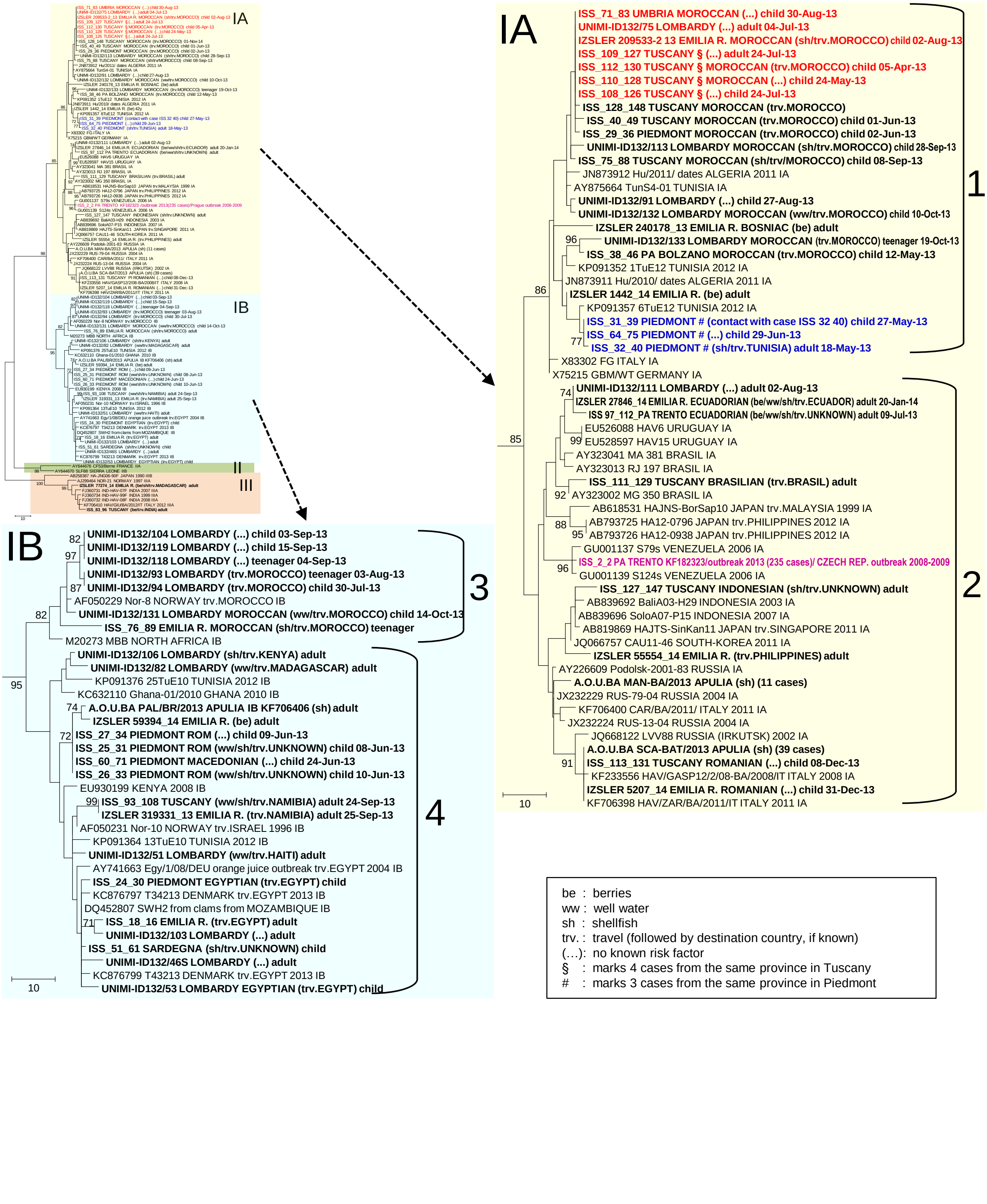
IA
IB
II
III
IA
ISS_71_83 UMBRIA MOROCCAN (...) child 30-Aug-13
UNIMI-ID132/75 LOMBARDY (...) adult 04-Jul-13
IZSLER 209533-2 13 EMILIA R. MOROCCAN (sh/trv.MOROCCO) child 02-Aug-13
ISS_109_127 TUSCANY § (...) adult 24-Jul-13
ISS_112_130 TUSCANY § MOROCCAN (trv.MOROCCO) child 05-Apr-13
ISS_110_128 TUSCANY § MOROCCAN (...) child 24-May-13
ISS_108_126 TUSCANY § (...) child 24-Jul-13
ISS_128_148 TUSCANY MOROCCAN (trv.MOROCCO)
ISS_40_49 TUSCANY MOROCCAN (trv.MOROCCO) child 01-Jun-13
ISS_29_36 PIEDMONT MOROCCAN (trv.MOROCCO) child 02-Jun-13
UNIMI-ID132/113 LOMBARDY MOROCCAN (sh/trv.MOROCCO) child 28-Sep-13
ISS_75_88 TUSCANY MOROCCAN (sh/trv/MOROCCO) child 08-Sep-13
JN873912 Hu/2011/ dates ALGERIA 2011 IA
AY875664 TunS4-01 TUNISIA IA
UNIMI-ID132/91 LOMBARDY (...) child 27-Aug-13
UNIMI-ID132/132 LOMBARDY MOROCCAN (ww/trv.MOROCCO) child 10-Oct-13
IZSLER 240178_13 EMILIA R. BOSNIAC (be) adult
96
UNIMI-ID132/133 LOMBARDY MOROCCAN (trv.MOROCCO) teenager 19-Oct-13
ISS_38_46 PA BOLZANO MOROCCAN (trv.MOROCCO) child 12-May-13
KP091352 1TuE12 TUNISIA 2012 IA
JN873911 Hu/2010/ dates ALGERIA 2011 IA
86
IZSLER 1442_14 EMILIA R. (be) adult
KP091357 6TuE12 TUNISIA 2012 IA
ISS_31_39 PIEDMONT # (contact with case ISS 32 40) child 27-May-13
ISS_64_75 PIEDMONT # (...) child 29-Jun-13
77
ISS_32_40 PIEDMONT # (sh/trv.TUNISIA) adult 18-May-13
1
X83302 FG ITALY IA
X75215 GBM/WT GERMANY IA
74
UNIMI-ID132/111 LOMBARDY (...) adult 02-Aug-13
IZSLER 27846_14 EMILIA R. ECUADORIAN (be/ww/sh/trv.ECUADOR) adult 20-Jan-14
ISS 97_112_PA TRENTO ECUADORIAN (be/ww/sh/trv.UNKNOWN) adult 09-Jul-13
EU526088 HAV6 URUGUAY IA
99
EU528597 HAV15 URUGUAY IA
85
AY323041 MA 381 BRASIL IA
AY323013 RJ 197 BRASIL IA
ISS_111_129 TUSCANY BRASILIAN (trv.BRASIL) adult
92
AY323002 MG 350 BRASIL IA
AB618531 HAJNS-BorSap10 JAPAN trv.MALAYSIA 1999 IA
88
AB793725 HA12-0796 JAPAN trv.PHILIPPINES 2012 IA
95
AB793726 HA12-0938 JAPAN trv.PHILIPPINES 2012 IA
GU001137 S79s VENEZUELA 2006 IA
ISS_2_2 PA TRENTO KF182323/outbreak 2013 (235 cases)/ CZECH REP. outbreak 2008-2009
96
GU001139 S124s VENEZUELA 2006 IA
ISS_127_147 TUSCANY INDONESIAN (sh/trv.UNKNOWN) adult
AB839692 BaliA03-H29 INDONESIA 2003 IA
AB839696 SoloA07-P15 INDONESIA 2007 IA
AB819869 HAJTS-SinKan11 JAPAN trv.SINGAPORE 2011 IA
JQ066757 CAU11-46 SOUTH-KOREA 2011 IA
IZSLER 55554_14 EMILIA R. (trv.PHILIPPINES) adult
AY226609 Podolsk-2001-83 RUSSIA IA
A.O.U.BA MAN-BA/2013 APULIA (sh) (11 cases)
JX232229 RUS-79-04 RUSSIA 2004 IA
KF706400 CAR/BA/2011/ ITALY 2011 IA
JX232224 RUS-13-04 RUSSIA 2004 IA
JQ668122 LVV88 RUSSIA (IRKUTSK) 2002 IA
A.O.U.BA SCA-BAT/2013 APULIA (sh) (39 cases)
91
ISS_113_131 TUSCANY ROMANIAN (...) child 08-Dec-13
KF233556 HAV/GASP12/2/08-BA/2008/IT ITALY 2008 IA
IZSLER 5207_14 EMILIA R. ROMANIAN (...) child 31-Dec-13
KF706398 HAV/ZAR/BA/2011/IT ITALY 2011 IA
2
10
IB
82
UNIMI-ID132/104 LOMBARDY (...) child 03-Sep-13
UNIMI-ID132/119 LOMBARDY (...) child 15-Sep-13
97
UNIMI-ID132/118 LOMBARDY (...) teenager 04-Sep-13
UNIMI-ID132/93 LOMBARDY (trv.MOROCCO) teenager 03-Aug-13
87
UNIMI-ID132/94 LOMBARDY (trv.MOROCCO) child 30-Jul-13
AF050229 Nor-8 NORWAY trv.MOROCCO IB
82
UNIMI-ID132/131 LOMBARDY MOROCCAN (ww/trv.MOROCCO) child 14-Oct-13
ISS_76_89 EMILIA R. MOROCCAN (sh/trv.MOROCCO) teenager
M20273 MBB NORTH AFRICA IB
3
UNIMI-ID132/106 LOMBARDY (sh/trv.KENYA) adult
UNIMI-ID132/82 LOMBARDY (ww/trv.MADAGASCAR) adult
KP091376 25TuE10 TUNISIA 2012 IB
95
KC632110 Ghana-01/2010 GHANA 2010 IB
74
A.O.U.BA PAL/BR/2013 APULIA IB KF706406 (sh) adult
IZSLER 59394_14 EMILIA R. (be) adult
72
ISS_27_34 PIEDMONT ROM (...) child 09-Jun-13
ISS_25_31 PIEDMONT ROM (ww/sh/trv.UNKNOWN) child 08-Jun-13
ISS_60_71 PIEDMONT MACEDONIAN (...) child 24-Jun-13
ISS_26_33 PIEDMONT ROM (ww/sh/trv.UNKNOWN) child 10-Jun-13
EU930199 KENYA 2008 IB
99
ISS_93_108 TUSCANY (ww/sh/trv.NAMIBIA) adult 24-Sep-13
IZSLER 319331_13 EMILIA R. (trv.NAMIBIA) adult 25-Sep-13
AF050231 Nor-10 NORWAY trv.ISRAEL 1996 IB
KP091364 13TuE10 TUNISIA 2012 IB
UNIMI-ID132/51 LOMBARDY (ww/trv.HAITI) adult
AY741663 Egy/1/08/DEU orange juice outbreak trv.EGYPT 2004 IB
ISS_24_30 PIEDMONT EGYPTIAN (trv.EGYPT) child
KC876797 T34213 DENMARK trv.EGYPT 2013 IB
DQ452807 SWH2 from clams from MOZAMBIQUE IB
71
ISS_18_16 EMILIA R. (trv.EGYPT) adult
UNIMI-ID132/103 LOMBARDY (...) adult
ISS_51_61 SARDEGNA (sh/trv.UNKNOWN) child
UNIMI-ID132/46S LOMBARDY (...) adult
KC876799 T43213 DENMARK trv.EGYPT 2013 IB
UNIMI-ID132/53 LOMBARDY EGYPTIAN (trv.EGYPT) child
4
10
be : berries
ww : well water
sh : shellfish
trv. : travel (followed by destination country, if known)
(…): no known risk factor
§ : marks 4 cases from the same province in Tuscany
# : marks 3 cases from the same province in Piedmont
